# Paediatric Congenital Enteropathies: Clinical and Histological Review

**DOI:** 10.3390/diagnostics15080946

**Published:** 2025-04-08

**Authors:** Francesca Arienzo, Isabella Giovannoni, Antonella Diamanti, Chiara Maria Trovato, Paola De Angelis, Chiara Imondi, Rita Alaggio, Paola Francalanci

**Affiliations:** 1 Pathology Unit, Bambino Gesù Children’s Hospital, IRCCS, 00165 Rome, Italy; 2 Hepatology, Gastroenterology and Nutrition Unit, Bambino Gesù Children’s Hospital, IRCCS, 00165 Rome, Italy; 3 Department of Medical-Surgical Sciences and Biotechnologies, Sapienza University of Rome, 00185 Rome, Italy

**Keywords:** enteropathies, structural disorders, immunomodulation disorders, VEO-IBD

## Abstract

Paediatric congenital enteropathies (PCEs) are a group of rare inherited diseases with a typical early onset in life. Prompt identification and treatment are crucial to avoid potentially fatal consequences. This review aims to provide a paradigmatic framework for clinical and histological identification of PCEs, with an emphasis on congenital conditions involving epithelial shape, trafficking and polarity, enteroendocrine function, immunomodulatory diseases, and extremely early onset inflammatory bowel illness. A proper classification is founded on histopathological characteristics and clinical parameters (such as consanguinity, anomalies in amniotic fluid, prenatal expression or early neonatal onset, stool appearance, persistence of symptoms despite fasting, and extra-intestinal manifestations, etc.). The increasing accessibility and convenience of genetic tests has also accelerated the identification of genes related to specific phenotypes of PCEs, improving the diagnostic and care pathway. As a “niche” pathology, PCEs are susceptible to misdiagnosis due to a limited awareness of these entities, and their identification requires extensive training and specialized facilities. The aim of our review is to emphasize the importance of an integrated approach, combining clinical, histological, and molecular analysis, to achieve a definitive diagnosis and guide the treatment.

## 1. Introduction

Paediatric congenital enteropathies (PCEs), also referred as congenital diarrhoeas and enteropathies, include a heterogeneous group of gastrointestinal disease that exhibit both phenotypic and genetic variability [1]. The most common clinical manifestation is a severe chronic diarrhoea during the first weeks of the life, accompanied by catastrophic dehydration and intestinal failure, referred to as congenital diarrhoea, making PCEs difficult to differentiate solely based on clinical features [2]. Consequently, to support treatment decisions that can prevent life-threatening complications, it is crucial to promptly detect and categorize PCEs using an integrated methodology. Most of these patients have a genetic cause, and the inability to tolerate oral nutrition and the need for prolonged parenteral nutrition (PN) are important clues [3]. However, the clinical phenotype is variable, and mild forms with later onset are described [1].

**Figure 1:**
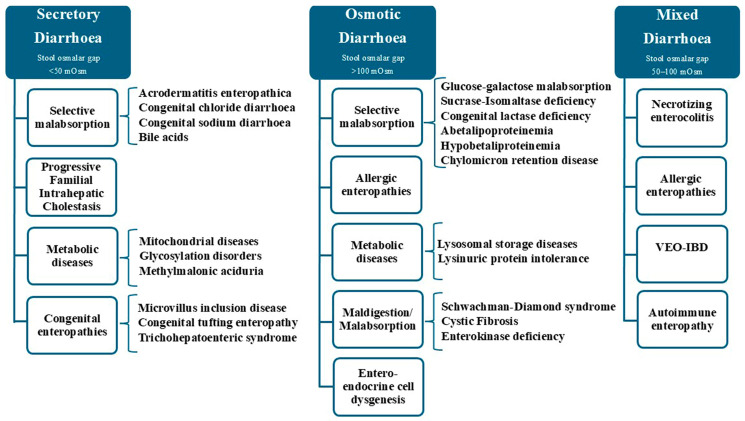
Overview of congenital diarrhoea. Abbreviation: VEO-IBD, very early onset inflammatory bowel disease.

The diagnostic approach starts from intravenous fluid replacement and PN. The response to fasting facilitates the diagnostic distinction between osmotic and secretory aetiologies of diarrhoea. Osmotic diarrhoea results from solute malabsorption, characterized by a high stool osmotic gap (>100 mOsm); these cases diminish with fasting. Secretory diarrhoea results from the active secretion of ions into the intestinal lumen, typically characterized by a modest osmotic gap in the stools (<50 mOsm). A stool sodium (Na^+^) value exceeding 80 mEq/L will confirm secretory diarrhoea. These types of diarrhoea persist after fasting. Lastly, diarrhoea that is obviously neither secretory nor osmotic, or has an intermediate stool osmotic gap (50–100 mOsm), has been referred to as “mixed”, not fully responsive to fasting [3]. Figure 1 illustrates an overview of congenital diarrhoeas of infancy.

The clinical and diagnostic algorithm (history and examination) provides a framework for assessment and prioritized testing (e.g., endoscopy and histopathology). A non-negligible aspect is the gestational and perinatal history: maternal polyhydramnios, prematurity, and failure to pass meconium suggest an intrauterine secretory process and a probable defect of the primary ion transporter. The regular passage of meconium followed by the onset of diarrhoea after a few days or even weeks suggests an epithelial disorder, whereas inflammatory forms generally have a later onset. If meconium passage was normal and diarrhoea resolves with fasting, osmotic diarrhoea is more likely, resulting from a primary defect in the digestive/absorptive system or, in some cases, an enteropathy. Other phenotypes include mixed presentations, protein-losing enteropathy, bloody diarrhoea, and selective fat malabsorption. Gathering a comprehensive family history to assess the presence or absence of consanguinity, recurrent miscarriages, or immune system disorders is crucial. Additionally, it is relevant to note phenotypic characteristics, such as dysmorphic features, hair anomalies, skin rashes, and perineal disease that may suggest a specific syndrome associated with enteropathy. There are also atypical phenotypes that appear later, beyond the neonatal period [4].

According to the diagnostic algorithm, most children with congenital diarrhoea will necessitate a biopsy of the small intestine. Histological sections stained with haematoxylin and eosin (H&E) facilitate the examination of the morphological characteristics of the intestinal mucosa, including architectural features (e.g., villus atrophy, villus height, crypt depth, goblet cells, assessment of inflammatory infiltrate), and the identification of glandular pathological features (e.g., cryptitis, crypt apoptosis). Histochemical stains and immunohistochemistry (e.g., Periodic Acid-Shiff [PAS], CD3, CD10, Ep-CAM) are supplementary techniques widely employed in daily practice to corroborate morphological findings. Even if electron microscopy (EM) is not usually performed, it is useful to evaluate specific epithelial structural disorders (e.g., presence of microvilli, epithelial tufts). As some congenital diarrhoeas may lack morphological characteristics features in the first months of life, endoscopic re-evaluation may be necessary when the child is older [4].

Nowadays, in many cases where a PCEs diagnosis is highly suspected but the exact aetiology is unknown or requires confirmation, next-generation sequencing with specific panels is the standard of care [4]. The increasing availability of molecular analyses has led to the identification of genetic defects in 90% of patients; the detection of new genetic mutations offered more insight into intestinal pathophysiology through genotype–phenotype correlations [1,5]. Whole-exome and whole-genome sequencing are employed in instances of complex phenotypes or when targeted gene panel sequencing fails to provide a conclusive genetic diagnosis [6,7].

From a pathophysiological point of view, PCEs can be classified into three main groups: deficiencies in nutrient/electrolyte transport, structural and functional abnormalities in absorption, and impairments of intestinal immune-related homeostasis [5]. This review provides a comprehensive overview of diagnostic methodology (clinical, pathological, and molecular studies) and therapeutic management of PCEs, specifically addressing the congenital disorders of intestinal differentiation, disorders of immunomodulation, and extremely early onset inflammatory bowel disease.

## 2. Intestinal Epithelial Structure and Function

The intestinal epithelium consists of a monolayer of cells that lines the luminal surface of the digestive tract. The small intestine is architecturally characterized by glands (crypts) and finger-like projections (villi). Various epithelial subtypes exist, including secretory cells such as goblet and enteroendocrine cells, as well as columnar absorptive cells known as enterocytes [8]. The surface epithelium of the small intestine is primarily responsible for fluid absorption and secretion, facilitated by transepithelial sodium transport and chloride secretion, respectively [9,10]. The apical membrane of enterocytes is covered by a well-organized network of microvilli that markedly expand the surface absorptive area (“brush border”) [11]. Microvilli contain significant sodium transporters, specific nutrition transporters, and digestive enzymes. The maintenance of transport proteins on microvilli is achieved through specialized polarized endocytic trafficking pathways in columnar epithelial cells.

## 3. Congenital Disorders Involving Epithelial Structure, Trafficking, Polarity, and Enteroendocrine Function

**Figure 2:**
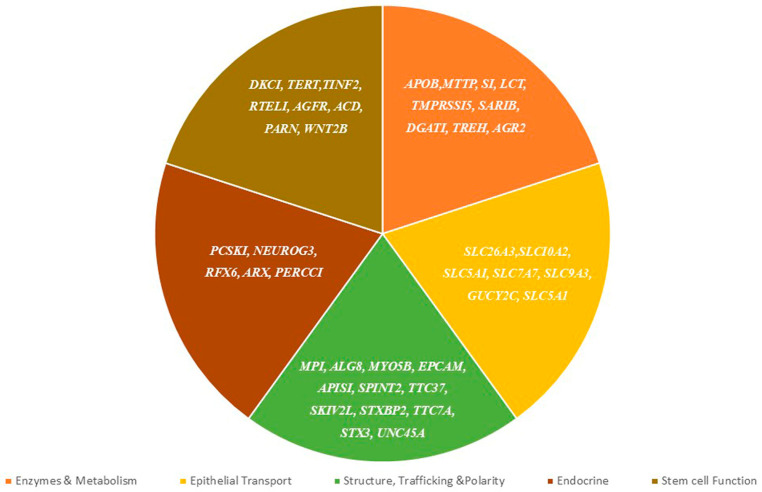
Genes in congenital disorders of the intestinal epithelia function.

Epithelial monogenic disorders encompass a variety of conditions distinguished by alterations in intestinal epithelial function, classified into five principal groups: epithelial transport; epithelial enzymes and metabolism; epithelial structure, trafficking, and polarity; enteroendocrine function; and epithelial stem cell function [12]. See Figure 2.

Congenital disorders of intestinal differentiation are described in detail in the following paragraphs.

### 3.1. Microvillus Inclusion Disease

Microvillus inclusion disease (MVID), initially described as microvillus atrophy, is an autosomal-recessive enteropathy characterized by refractory secretory diarrhoea (typically ranging between 150 and 300 mL/kg/day) that occurs in the first days of life (early onset form) or in the first 2–3 months (late-onset form) [13,14].

There is wide variation in clinical phenotype: affected infants manifest developmental delay, liver and kidney damage, and, in up to one-third of cases, may develop early onset cholestasis and liver cirrhosis. In addition, cases of associated Fanconi syndrome have also been reported [15].

The underlying pathogenetic mechanism consists of a disturbance in the trafficking machinery of the intestinal epithelium that leads to a polarity-associated disorder. Most patients have been found to have a mutation in the Myosin-Vb (*MYO5B*) gene, located on chromosome 18q21.1, encoding an actin filament-based motor protein that binds select GTPase RAB proteins [16]. *MYO5B* is also required for proper localization of the bile salt export pump ABCB11 at the apical/canalicular plasma membrane of hepatocytes; its deficiency impairs the bile salt export pump function in the canalicular membrane, contributing to cholestasis [17,18].

Numerous distinct homozygous and compound heterozygous mutations of *MYO5B* have been found over the years. Specific mutations in *MYO5B* may influence both early and late onset, as well as diverse clinical presentations [19]. Chronic diarrhoea is linked to familial hemophagocytic lymphohistiocytosis type 5 and metabolic acidosis in patients with syntaxin-binding protein 2 (*STXBP2/MUNC18-2*) mutation and to neurological impairments in patients with syntaxin 3 (*STX3*) mutation, respectively [20,21,22]. MVID-like congenital diarrhoea in osteo-oto-hepato-enteric syndrome patients with biallelic mutations in the unc-45 myosin chaperone A (*UNC45A*) gene has been previously documented [23].

Macroscopic endoscopic examination of the small bowel and colon is usually normal. Small bowel biopsy is typically characterized by severe villus atrophy and mild to moderate crypt hyperplasia, with a normal number of IELs [19].

PAS staining shows the absence of a distinct brush border and the presence of diffuse PAS-positive inclusions at the apex of the enterocytes. Immunohistochemical staining with antibodies anti-CD10 and anti-villin shows an irregular apical border staining, corresponding to abnormal subapical localization of brush border components [24].

**Figure 3:**
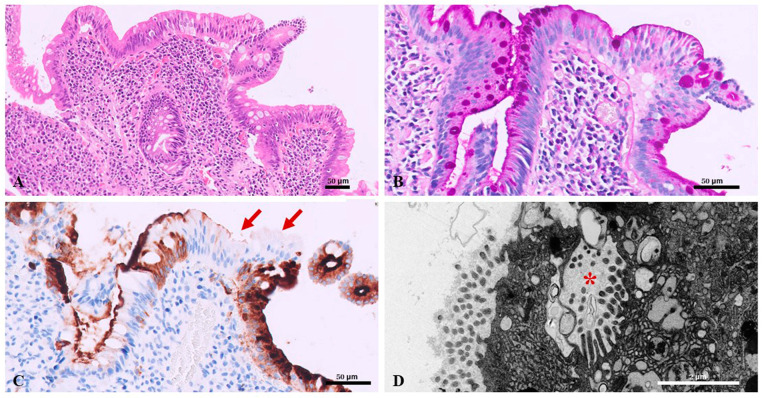
Microvillus inclusion disease. Duodenal biopsy shows (**A**) severe villous atrophy H&E, 10×; (**B**) absence of a distinct brush border and the presence of diffuse PAS-positive inclusions at the apex of the brush border, 40×; (**C**) immunohistochemical staining with antibodies anti-CD10 shows an absent apical border staining (arrows), 40×; (**D**) ultrastructural study reveals absent brush border microvilli and the presence of microvillus inclusion (asterisk) in the apical cytoplasm of enterocytes. Scale bars = 50 µm in (**A**–**C**), and 2 µm in (**D**).

Pathognomonic ultrastructural findings include shortening or absence of apical microvilli and microvillus inclusions in mature enterocytes located toward the apex of the enterocytes. The main histological and ultrastructural features are shown in Figure 3.

Individuals with MVID require lifelong nutritional support through PN. PN is often challenging due to profound electrolyte and water losses and early complications such as recurrent central venous catheter-related bloodstream infections, as well as cholestatic liver disease. However, in patients with MVID, cholestasis is predominantly associated with genetic disorder rather than extended PN. Some patients progress to bowel transplantation, burdened by a high risk of complications such as cholestasis and immunosuppression-related infections [4]. Patients with late-onset MVID and a mild clinical profile can tolerate enteral feeding, hence decreasing the necessity for parenteral nutrition to once or twice weekly [25].

### 3.2. Congenital Tufting Enteropathy

Congenital tufting enteropathy (CTE), also known as epithelial dysplasia, is a rare autosomal recessive disease that manifests within the first month of life as intractable secretory diarrhoea with varying severity and intestinal failure [26].

Since 2008, when the homozygous mutation in the Epithelial Cell Adhesion Molecule (*EpCAM*) gene was first identified as a causative gene, more than one hundred *EpCAM* mutations have been reported [27,28,29]. The *EpCAM* gene, located on chromosome 2p21, encodes for a conserved type I transmembrane superficial glycoprotein expressed in the tight junctions and basement membrane of most normal epithelial cells. A genotype–phenotype correlation has also been identified: patients carrying the c.499dupC *EpCAM* mutation are reported to have a more severe disease phenotype [30].

In more than seventy percent of cases, homozygous or compound heterozygous mutations in the *EpCAM* gene are identified and patients display isolated digestive symptoms [26]. A syndromic form, characterized by symptoms such as punctate keratitis and choanal atresia, is associated with mutations in Serine Peptidase Inhibitor Kunitz Type 2 (*SPINT2*) [30]. *SPINT2*, located on chromosome 19q13.2, encodes a transmembrane protein with two extracellular Kunitz domains that inhibit a variety of serine proteases, such as prostasin, which is important for epithelial barrier formation [31].

The appropriate site for histological examination is the duodenum. Histological features can be subtle and include non-specific features such as villus blunting/atrophy, crypt hyperplasia, an increased number of mitotic figures in the crypts, and basement membrane irregularities in the absence of inflammation [32]. The hallmark “tufting appearance” consists of epithelial cell crowding with apical displacement of nuclei with rounding of the cytoplasmic membrane [32]. Ancillary immunohistochemistry is a useful diagnostic tool: patients with *EpCAM* mutations lack Ep-CAM (MOC-31) staining, which is similar to normal controls in patients with *SPINT2* mutations [33].

**Figure 4:**
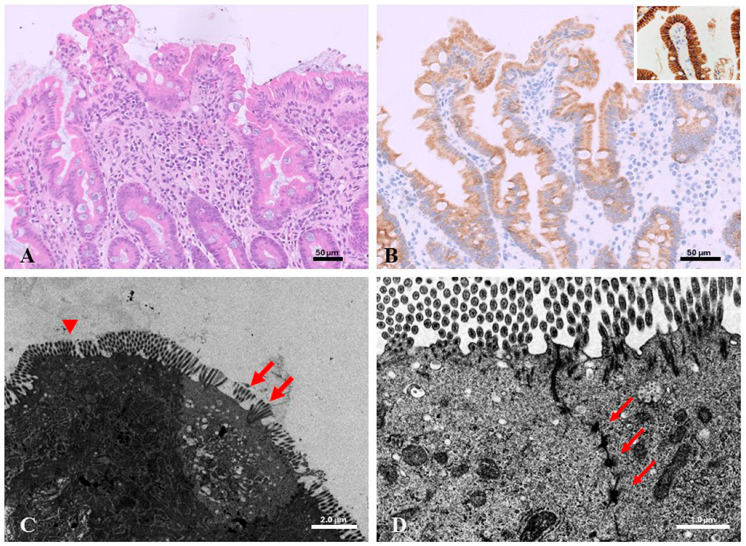
Congenital tufting enteropathy. Duodenal biopsy (**A**) villous blunting, crypt hyperplasia, and mild inflammation, H&E, 20×; (**B**) Ep-CAM (MOC-31) staining, 20×; in the inset, a normal control showing strong cytoplasmatic positivity of Ep-CAM, 40×; (**C**) the surface with evidence of tufts (arrows) compared with normal parallel disposition of microvilli (arrowhead); (**D**) desmosomes increased in length (arrows). Scale bars = 50 µm in (**A**,**B**), 2.0 µm in (**C**), and 1.0 µm in (**D**).

EM shows evidence of tufts and desmosomes increased in length and in number. The main histological and ultrastructural features are shown in Figure 4.

Despite some phenotypic variability, most patients require PN to maintain adequate nutritional status and growth [34].

### 3.3. IDEDNIK Syndrome

Intellectual disability, enteropathy, deafness, neuropathy, ichthyosis, and keratodermia (IDEDKIN) syndrome, previously referred to as mental retardation (M)EDNIK syndrome, is characterized by severe and persistent diarrhoea within the first week of life with severe dehydration and salt-wasting, requiring prompt correction of acid–base and electrolyte imbalances, as well as the initiation of PN [35]. Another hallmark of IDEDKIN syndrome is a defect in copper transport, resulting in reduced copper levels and copper accumulation in the liver, similar to what is observed in Wilson’s disease [36].

Adaptor Related Protein Complex 1 Subunit Sigma 1 (*AP1S1*) is a protein-coding gene on chromosome 7q22.1 that is associated with this syndrome, and it encodes a protein that is a component of the clathrin coat-assembly complex [37]. It is important in trafficking due to connecting clathrin or other coat proteins to receptors in coated vesicles, selectively sorting cargo among the cell membrane, trans-Golgi network, and endosomal compartments [37,38]. Proteins responsible for maintaining adhesion include EpCAM and E-cadherin, while those that form tight junctions include the claudins, occludin, and zonula occludens-1 (ZO-1) [39]. Animal models have shown that when ZO-1 and claudin-3 become mislocalized to the basolateral membrane, tight junctions stop working. This is thought to be the causative mechanism of enteropathy in IDEDNIK Syndrome [39]. Histological sections of the duodenum showed mild villous blunting and enterocytes with cytoplasmic vacuoles. CD10 immunostaining highlighted the disruption of the brush border, and Ep-CAM (MOC-31) displayed a membranous pattern of expression, similar to the normal control. EM showed scattered enterocyte cells with shortened and disrupted apical microvilli [40].

Supportive care involves dietary adjustments, feeding therapy, and enteral or parenteral supplementation for enteropathy [35].

### 3.4. Enteroendocrine Cell Dysgenesis

Enteroendocrine cells are specialized secretory lineage cells that, although constituting a minority of the intestinal epithelial population, collectively represent the biggest endocrine organ in the body. Neurogenin 3 (*NEUROG3*), Proprotein Convertase Subtilisin/Kexin Type 1 (*PCSK1*), and Proline and Glutamate Rich with Coiled Coil 1 (*PERCC1*) genes play a pivotal role in normal enteroendocrine cell development and differentiation [41,42,43]. Enteroendocrine cell dysgenesis (ECD) is an autosomal recessive inherited disorder, clinically presenting with malabsorption and severe secretory diarrhoea from birth [41]. These disorders result in the depletion of enteroendocrine cells, underscoring their critical role in normal fluid and nutrient absorption.

The homozygotic germline mutation of *NEUROG3* is the first causative molecular alteration identified in ECD [44]. The *NEUROG3* gene, located on chromosome 10q22.1, encodes a transcription factor that regulates the developmental pathway of gut and pancreatic epithelial stem cells destined to become endocrine cells [44]. Individuals with *NEUROG3* mutations may also present with extra-intestinal symptoms, most often insulin-dependent diabetes, with onset ranging from infancy to later childhood, and, rarely, hypogonadotropic hypogonadism [45].

Clinical suspicion of ECD can be proven through immunohistochemistry for chromogranin, which demonstrates the absence of enteroendocrine cells in the small intestine or colon [46]. Histological findings are not always clear cut and may include variable villous atrophy, normal surface epithelium, and minimal inflammation in the lamina propria. It is important to remember that individuals with autoimmune enteropathy (AIE) could show a negative chromogranin stain, but those with AIE, conversely from EED, also show a loss of goblet and Paneth cells [47]. EM shows the absorptive columnar cells with a normal brush border (“microvilli”) and the absence of microvillus inclusions.

Currently, PN seems to be the most suitable therapy, supplemented with varying amounts of enteral nutrition based on each patient’s tolerance [4].

### 3.5. Tricho-Hepato-Enteric Syndrome

Tricho-hepato-enteric syndrome (THES), referred to as phenotypic or syndromic diarrhoea, is a rare congenital enteropathy characterized by a triad of chronic diarrhoea starting in the first 6 months of life, facial dysmorphism, and hair abnormalities (woolly, thickened hair prone to breakage known as trichorrhexis nodosa) [48]. THES is caused by a mutation in either the SKI3 subunit of the superkiller complex (*SKIC3*), formerly the tetratricopeptide repeat domain-containing protein 37 (*TTC37*) mapping on chromosome 5q1, or the SKI2 subunit of the superkiller complex (*SKIC2*), formerly the SKI2-like RNA helicase (*SKIV2L*) mapping on chromosome 6p21.33. These are two genes that code for parts of the human SKI complex that breaks down RNA [49].

Many children present also with intrauterine growth restriction, short stature, immunodeficiency, skin abnormalities, and liver disease [50]. Non-specific liver dysfunction is common and may significantly impact prognosis, with mortality in the first five years of life often due to cirrhosis and liver failure [51]. Approximately 50% of affected individuals have mild intellectual disability [50]. Less common findings include congenital heart defects and platelet abnormalities. Immune abnormalities are diverse and frequently observed, including hypogammaglobulinemia requiring periodic immunoglobulin supplementation. The switched memory B-lymphocyte count is typically very low, IFN-γ production by T and NK cells is impaired and associated with a reduced degranulation of NK cells, and T-cell proliferation is frequently abnormal [52].

Histopathology of the small bowel shows normal mucosa in about 25% of cases, mild villous atrophy in 50% of cases, and subtotal villous atrophy in 25% of cases [1].

To promote optimal weight gain and linear growth, most children initially require PN combined with a semi-elemental diet, if tolerated [50]. The inheritance of THES is autosomal recessive, highlighting the importance of parental genetic counselling.

## 4. Disorders of Immunomodulation

AIE is a rare disorder characterized by severe and prolonged diarrhoea, weight loss due to malabsorption, and immune-mediated damage to the intestinal mucosa [53]. It generally occurs in infants and young children but is rarely seen in adults. Anti-enterocyte antibodies are identified in the majority of cases, and their presence, even if variable, can help to confirm the diagnosis [54]. AIE seems to stem from the disruption of gastrointestinal humoral and immunological activities [55]. Two main syndromes are fundamentally caused by genetic defects that result in T-cell hyperactivation: (i) Immune Dysregulation, Polyendocrinopathy, Enteropathy, and X-linked syndrome (IPEX), caused by mutations in *forkhead box P3* (*FOXP3*) gene, and (ii) Autoimmune Polyendocrinopathy, Candidiasis, and Ectodermal Dystrophy syndrome (APECED), also known as autoimmune polyglandular syndrome type-1 (APS-1), caused by variants in the autoimmune regulator (*AIRE*) gene [56,57]. AIE may be linked with various primary immunodeficiencies, including common variable immunodeficiency and hypogammaglobulinemia, the latter being the most frequently reported condition [53]. In AIE, abnormal expression of self-antigens on epithelial cells activates CD4+ T lymphocytes, leading to downstream effects that culminate in the destruction of enterocytes through apoptosis or other cytotoxic mechanisms [58]. Autoantibodies may target goblet cells, enterocytes, and the intestinal brush border. Autoantibodies targeting AE75, an intestinal antigen crucial for maintaining tight junctions and cytoskeletal integrity, result in increased intestinal permeability [59]. Approximately 50% of patients with otherwise typical AIE manifestations, including female patients, have no detectable mutations in *FOXP3*, earning them the designation of IPEX-like [60]. These patients have been found to harbour mutations in a variety of immune regulatory genes, including cluster of differentiation 25 (CD25), signal transducer and activator of transcription 5b (*STAT5b*), cytotoxic T-lymphocyte-associated protein 4 (*CTLA-4*), Itchy E3 Ubiquitin Protein Ligase (*ITCH*), lipopolysaccharide responsive and beige-like anchor protein (*LRBA*), and others [60]. The conditions associated with AIE are summarized in Table 1.

The salient histopathologic features are most prominent in the small intestine: villous blunting, crypt hyperplasia, mononuclear cell inflammatory expansion of the lamina propria with IELs, crypt apoptosis similar to those seen in intestinal graft-versus-host disease, and absence of Paneth cells, goblet cells, or both. Oesophagus, stomach, and colon are frequently also involved [61]. Table 2 summarizes the histopathology and grade of the lesions considered for the final diagnosis.

**Figure 5:**
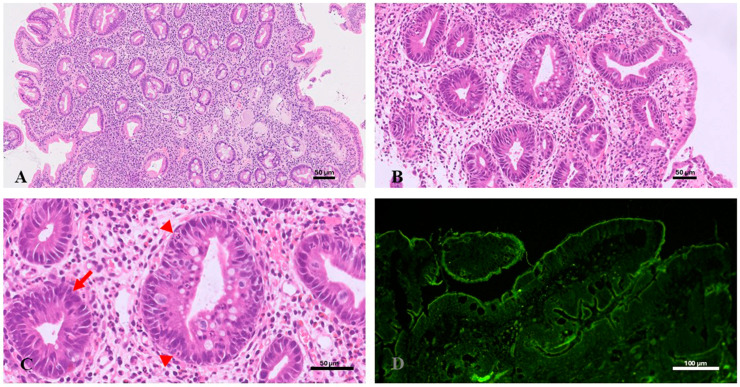
Autoimmune enteropathy. Duodenal biopsy (**A**) villous blunting, H&E, 10×; (**B**) crypt hyperplasia and mononuclear cell inflammatory expansion of the lamina propria with intraepithelial lymphocytosis, 20×; (**C**) crypt apoptosis (arrowheads), absence of Paneth cells, and decreasing goblet cells (arrow), 40×; (**D**) indirect immunofluorescence using serum from the patient against normal human small bowel: diffuse linear staining of IgG along the apical border of the villi, 20×. Scale bars = 50 µm in (**A**–**C**) and 100 µm in (**D**).

Depletion of goblet and Paneth cells, generally not observed in other inflammatory intestinal diseases, and increased apoptosis in the base of crypts may address a diagnosis of AIE [62]. In the case of suspicion, an additional fresh biopsy should be sent to pathology for indirect immunofluorescence using the patient’s serum to detect the presence of anti-enterocyte antibodies. Positive fluorescent staining on indirect immunofluorescence results in a linear pattern along the apex and basolateral border of the enterocyte. The antibodies are predominantly IgG and have been described as complement-fixing, though IgM and IgA have also been reported [1]. The main histological features are shown in Figure 5.

## 5. Very Early Onset Inflammatory Bowel Disease

Very early onset inflammatory bowel disease (VEO-IBD) refers to IBD-like symptoms in children under six years, encompassing a heterogeneous spectrum of disorders, characterized by gastrointestinal and extra-gastrointestinal manifestations [63]. VEO-IBD can be further subclassified into infantile IBD, for children diagnosed before two years of age, and neonatal IBD, for those diagnosed before 28 days of life [64]. VEO-IBD includes classic IBD, [Crohn’s disease (CD) and ulcerative colitis (UD)], and monogenic disorders in 20–30% of cases, often involving genes dysregulated in primary immunodeficiencies [65]. Unlike older-onset IBD patients, children with VEO-IBD have a higher rate of IBD-unclassified, a positive family history, a more aggressive clinical course, and a resistance to conventional therapy for IBD [66]. In cases of monogenic diseases, the histological lesions show a more severe and extensive phenotype than older-onset IBD [67]. Current diagnostic approaches are based on clinical suspicion, laboratory, radiological, and histological evaluation, followed by molecular analysis confirmation.

Monogenic VEO-IBDs may have unusual features including the association of IBD-like presentation with primary immunodeficiencies, such as common variable immunodeficiency, Wiskott–Aldrich syndrome, chronic granulomatous disease, and nuclear factor-kappa B Essential Modulator (NEMO) syndrome. The implicated genes frequently participate in pro-inflammatory immunological pathways, indicating prospective targets for pharmacological interventions. In fact, in an increasing number of cases, these monogenic immunodeficiencies can be treated with targeted pharmacologic agents and other therapies, such as hematopoietic stem cell transplantation [68].

**Figure 6:**
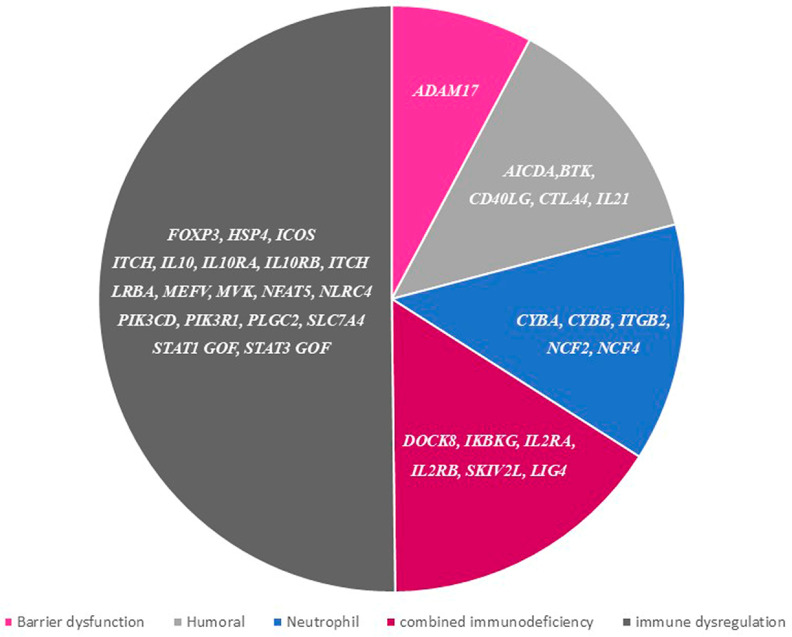
Aetiology of monogenic very early onset inflammatory bowel disease.

To date, a useful classification of the monogenic aetiologies of VEO-IBD includes six main (and sometimes overlapping) categories: (1) general immune dysregulation, (2) T- and B-cell defects, (3) phagocytic defects, (4) hyper- and auto-inflammatory conditions, (5) epithelial barrier dysfunction, and (6) other conditions [69]. Recent advances in molecular technology, such as whole-exome sequencing, have allowed the discovery of more than 50 genes and pathways associated with VEO-IBD [70]. Figure 6 presents the landscape of current genes associated with monogenic VEO-IBD.

The physical exam remains a useful tool in evaluating VEO-IBD patients to raise suspicion of a monogenic disorder. Monogenic IBD patients usually have other health issues and/or extra-intestinal symptoms. However, it has been reported that monogenic disorders can also present with isolated gastrointestinal involvement [4]. There is a set of associated anomalies that may be frequently in monogenic disorders, including dysmorphic features, hepatomegaly, splenomegaly, atopic dermatitis, hyperkeratosis, albinism, and epidermolysis bullosa. For example, a variety of severe perianal disease, folliculitis, and arthritis in young patients presenting within the first few months of life is suggestive of interleukin-10 signalling defects [71]. Features of common variable immunodeficiency, such as *LRBA* or *CTLA4* deficiency, include recurrent infections, various autoimmune and endocrine disorders, and organomegaly. Patients with IPEX syndrome typically manifest enteropathy associated with type 1 diabetes mellitus, eczema, food allergies, and a variety of other autoimmune manifestations. In the NEMO syndrome, often ectodermal dysplasia is associated with enteropathy. In patients with A disintegrin and metalloprotease 17 (ADAM17) deficiency, hair and/or nail abnormalities are also common observed. Finally, in some cases, monogenic VEO-IBD is also associated with either hemophagocytic lymphohistiocytosis or macrophage-activating syndromes, and some patients may have an increased risk of developing neoplasms (such as diffuse large B-cell lymphoma in interleukin-10 signalling defects) [66].

To date, endoscopy with multiple biopsies for histological characterization represents the diagnostic gold standard for VEO-IBD [72]. Despite the predominance of colonic inflammation, it is essential to always perform an endoscopic evaluation of the small bowel and upper digestive tract, considering the evolving behaviour of VEO-IBD. The small bowel endoscopic evaluation using wireless capsule endoscopy is, however, challenging in smaller children weighing less than 10 kg, due to the technical difficulty of wireless capsule endoscopy positioning [66].

Four histologic patterns are described in gastrointestinal biopsies from VEO-IBD [65].

(1) A CD-like pattern characterized in the small bowel by irregular villus morphology, irregular inflammatory infiltrate, crypt hyperplasia, focal cryptitis, granulomas, lymphoid hyperplasia, and aphthous lesions over lymphoid aggregates. In the colon, it presents with a discontinuous and transmural mixed inflammatory infiltrate, deep ulceration of the mucosa, and crypt abscess formation and granulomas.
(2) A UC-like pattern characterized by a continuous marked mixed inflammatory infiltrate, moderate-to-severe architectural gland atrophy/distortion, Paneth cell metaplasia, muco-depletion, foci of cryptitis, pseudo-abscesses, ulcerations, and focal detachment of superficial epithelium.
(3) An enterocolitis-like pattern characterized in the small bowel by widespread villus atrophy and by a preserved glandular architecture, extensive detachment of superficial colonic epithelium, widespread lymphocytic and eosinophilic infiltrate, exudate and mucosal friability, oedema, and ischemic ulcers. Cytomegalovirus superinfection may also occur in UC and CD.
(4) An apoptotic pattern characterized by scattered glands and severe glandular atrophy, increased mononuclear cells within the lamina propria, apoptotic cell death, extensive apoptosis, gland dropout, and “exploding crypts”.

**Figure 7:**
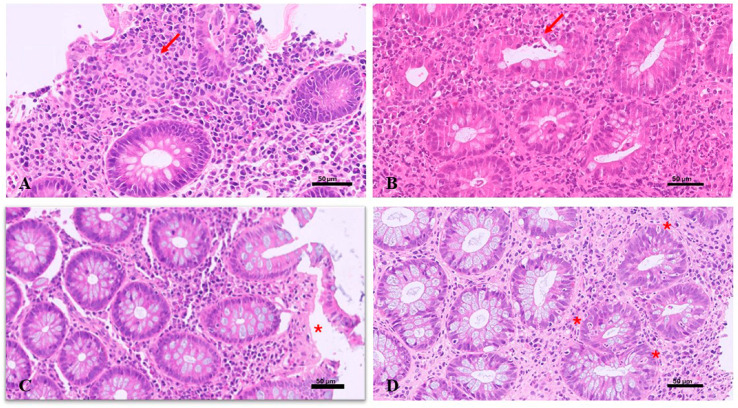
Very early onset inflammatory bowel disease. Colon biopsy. (**A**) CD-like pattern: polymorphous inflammatory infiltrate with epithelioid granuloma (arrow), H&E, 20×. (**B**) UC-like pattern: polymorphous inflammatory infiltrate with plasma cells and eosinophils with moderate glandular distortion, cryptitis, and initial cryptic abscess (arrow), H&E, 20×. (**C**) Enterocolitis-like: marked homogeneous inflammatory infiltrate with preserved glandular architecture and mucosal pseudo-detachment (asterisk), H&E, 20×. (D) Apoptotic-like pattern: glandular distortion, dense eosinophilic infiltrate in the lamina propria, and apoptotic bodies (asterisk), H&E, 20×. Scale bars = 50 µm in (**A**–**D**).

Figure 7 shows the four histological patterns described above.

## 6. Therapeutic Outlook

From a therapeutic point of view, almost all PCEs require nutritional support to maintain nutritional and electrolyte balance. The primary goal is to integrate the maximum tolerated amount of enteral nutrition and oral feeding with ongoing PN support. The long-term aim is to achieve at least partial intestinal autonomy, although many patients will continue to require PN [73].

Several forms of PCEs, nevertheless, can be favourably treated with specific therapeutic strategies, according to the underlying aetiology, which can improve or reverse the associated intestinal failure, thus reducing or stopping the dependence on PN. Specific nutritional and non-nutritional strategies are available [73]:

(1) Nutritional strategies: exclusion of specific nutrients from the diet (e.g., in glucose-galactose malabsorption); electrolytes support (e.g., in congenital chloride diarrhoea); and low-fat diet, medium-chain triglycerides supplementation, high protein intake, vitamins, and electrolyte supplements in diseases associated with protein-losing enteropathy.
(2) Non-nutritional strategies: hematopoietic stem cell transplantation, intestinal transplantation, steroids, immunosuppressants, and biologics can be employed in the defects of intestinal homeostasis according to the specific aetiology.

## 7. Conclusions

PCEs are a rare and heterogeneous group of inherited disorders that typically present early in life. Some disorders present with little or no chronic mucosal inflammation, while others are characterized by varying degrees of mucosal inflammation, such as VEO-IBD or primary immunodeficiency with intestinal involvement that primarily affects immune cell function. The genetic basis of PCEs is now increasingly well-defined, and information on specific disorders as outlined in this review continues to grow. The clinical diagnostic algorithm (history and examination) provides a framework for assessment and prioritized testing. This structured approach ensures that the most relevant information is gathered to guide clinical decision-making. Close collaboration between paediatric immunologists, gastroenterologists, pathologists, and geneticists is essential to optimize the diagnosis and management of PCEs. The subsequent frontier in this domain is to convert these novel genetic and molecular discoveries into targeted therapies that can profoundly impact the lives of PCEs patients.

In conclusion, Table 3 provides a summary of the histological and clinical features and therapeutic management of PCEs discussed in this review.

## Figures and Tables

**Table 1:** Conditions associated with autoimmune enteropathy: causative genetic mutations, the mechanism of action of the mutation, the main histopathological features, and other clinical features. Abbreviations: ALPS-like, autoimmune lymphoproliferative syndrome-like; IELs, Intraepithelial Lymphocytes.

Condition	Mutation	Mechanism	Histopathology	Other Clinical Features
IPEX	FOXP3	Impaired Treg	Villus atrophy, enteropathy	Endocrinopathies, dermatitis
APECED/ APS-1	AIRE	Impaired central tolerance	Villus blunting, absence of enteroendocrine cells	Candidiasis, endocrine dysfunction
IPEX-LIKE Disorders				
CD25 deficiency	IL2Rα	Impaired Treg functions	Villus atrophy	Recurrent infections
STAT 5b deficiency	STAT5b	Impaired Treg functions	Villus atrophy	Growth failure, immunodeficiency, pulmonary disease
CTLA-4 haploinsufficiency	CTLA-4	Abnormal Treg function	Villus atrophy, ALPS-like proliferation	Recurrent infections, cytopenia
ITCH mutation	ITCH	Abnormal T cell activity	IBD-like	Dysmorphism, short stature, pulmonary disease
LRBA deficiency	LRBA	Abnormal function of CTLA-4	Villus atrophy, IELs	Recurrent infections, lymphoproliferative disorders

Adapted from Chen, C.B. et al. 2020 [60].

**Table 2:** Histopathological parameters in autoimmune enteropathy. Abbreviations: IELs, Intraepithelial Lymphocytes.

Criteria	Grade
Villus atrophy	normal/partial villus atrophy, subtotal total atrophy
Goblet cells	present, reduced, absent
Inflammatory infiltration	absent-mild-moderate-severe, IELs evaluated with CD3
Type of inflammatory cells	lymphocytes, plasma cells, neutrophils, eosinophils
Glandular pathological features	cryptitis, crypt abscesses, necrosis, apoptosis
Crypt epithelial apoptosis	>1/10 crypts

**Table 3:** Paediatric enteropathies: clinical, diagnostic, and therapeutics essentials. Abbreviations: EM, Electron Microscopy; EEC, enteroendocrine cells; GI, gastrointestinal; EEN, Exclusive Enteral Nutrition; EN, Enteral Nutrition; IELS, Intraepithelial Lymphocytes; IF, immunofluorescence; PN, parenteral nutrition.

Disease Type	Main Clinical Features	Diagnosis		Therapeutics
		Histopathology	Genetics	
*Microvillus inclusion disease*	intractable secretory diarrhoea ▪ Early onset form ▪ late-onset form	severe villus atrophy EM microvillous inclusion	*MYO5B* *STXBP2* *STX3* *UNC45A*	PN; bowel transplantation; enteral feeding *plus* PN (1/2 per week) in late-onset form
*Congenital Tufting enteropathy*	intractable secretory diarrhoea ▪ classic form ▪ syndromic form	villus atrophy tufting appearance EM evidence of tufts; altered desmosomes	*EpCAM* *SPINT2*	PN combined with a semi-elemental diet, if tolerated
*IDEDNIK Syndrome*	intellectual disability, enteropathy, deafness, neuropathy, ichthyosis, and keratodermia	mild/subtotal villus atrophy	*AP1S1*	oral zinc acetate or oral zinc sulfato therapy; dietary modifications, feeding therapy, and parenteral supplementation; hearing aids; neurological treatment; supportive therapies
*Enteroendocrine cell dysgenesis*	malabsorption and severe diarrhoea	absence of intestinal EEC (chromogranin negative) EM normal brush border	*NEUROG3; ARX; PCSK1*; *RFX6* *PERCC1*	PN and minimal enteral feeding, if tolerated
*Trichohepatoenteric syndrome (Phenotypic or Syndromic Diarrhoea)*	chronic diarrhoea starting in the first 6 months of life, facial dysmorphism, and hair abnormalities	mild and subtotal villus atrophy	*TTC37* *SKIV2L*	PN combined with a semi-elemental diet, if tolerated
*Autoimmune enteropathy*	severe and prolonged diarrhoea, weight loss due to malabsorption, and extra-GI autoimmune disease	villous blunting; crypt hyperplasia; IELs; crypt apoptosis; absence of Paneth cells IF	*FOXP3; AIRE; IL2Rα; STAT5b; CTLA-4; ITCH LRBA*	immunosuppressive and immunomodulatory drugs combined with supportive PN and EN, if necessary
*Very early onset inflammatory bowel disease*	GI and extra-GI symptoms	CD-like pattern; UC-like pattern; Enterocolitis-like pattern; apoptotic pattern	Multiple genes (Figure 6)	immunosuppressive and immunomodulatory drugs combined with supportive EN or, in some selected cases, EEN targeted pharmacologic agents and other therapies, such as hematopoietic stem cell transplantation, in selected cases

## Data Availability

No new data were created or analysed in this study. Data sharing is not applicable to this article.

## Abbreviations

The following abbreviations are used in this manuscript:

*ADAM17*	A disintegrin and metalloprotease 17
AIE	Autoimmune enteropathy
*AIRE*	Autoimmune Regulator
AP1SP1	Adaptor Related Protein Complex 1 Subunit Sigma 1
APECED	Polyendocrinopathy, Candidiasis, and Ectodermal Dystrophy syndrome
APS-1	autoimmune polyglandular syndrome type-1
CD	Crohn’s Disease
CD25	cluster of differentiation 25
CTE	Congenital Tufting Enteropathy
*CTLA-4*	cytotoxic T-lymphocyte-associated protein 4
EED	Enteroendocrine Cell Dysgenesis
EM	Electron Microscopy
*EpCAM*	Epithelial Cell Adhesion Molecule
H&E	Haematoxylin and Eosin
IDEDNIK	Intellectual disability, enteropathy, deafness, neuropathy, ichthyosis, and keratodermia
IELs	Intraepithelial Lymphocytes
IPEX	Immune Dysregulation, Polyendocrinopathy, Enteropathy, and X-linked syndrome
*ITCHY*	Itchy E3 Ubiquitin Protein Ligase
*LRBA*	Lipopolysaccharide-Responsive and Beige-like Anchor protein
MEDNIK	Mental retardation, enteropathy, deafness, neuropathy, ichthyosis, and keratodermia
MVID	Microvillus Inclusion Disease
*MYO5B*	Myosin-Vb
*NEMO*	Nuclear factor-kappa B Essential Modulator
*NEUROG3*	Neurogenin 3
PAS	Periodic Acid-Shiff
PCEs	Paediatric Congenital Enteropathies
*PCSK1*	Proprotein Convertase Subtilisin/Kexin Type 1
*PERCC1*	Proline and Glutamate Rich with Coiled Coil 1 (*PERCC1*)
PN	Parenteral Nutrition
*SKIC2*	SKI2 subunit of the superkiller complex
*SKIC3*	SKI3 subunit of the superkiller complex
*SKIV2L*	SKI2-like RNA helicase
*SPINT2*	Serine Peptidase Inhibitor Kunitz Type 2
*STX3*	syntaxin 3
*STAT5b*	signal transducer and activator of transcription 5b
*STXBP2*	syntaxin-binding protein 2
THES	Tricho-hepato-enteric Syndrome
*TTC37*	tetratricopeptide repeat domain-containing protein 37
UC	Ulcerative Colitis
*UNCA-45*	unc-45 myosin chaperone A
VEO-IBD	Very Early Onset Inflammatory Bowel Disease
ZO-1	Zonula occludens-1
